# The effects of prehospital system delays on the treatment efficacy of STEMI patients

**DOI:** 10.1186/s13049-019-0616-4

**Published:** 2019-04-08

**Authors:** Magdalena Żurowska-Wolak, Patryk Piekos, Jacek Jąkała, Marcin Mikos

**Affiliations:** 1 Jagiellonian University Medical College, Faculty of Health Sciences, Division of Emergency Medical Services, Kraków, Poland; 2 Department of Anatomy, Jagiellonian University Medical College, Kraków, Poland; 3 Cracow Center of Invasive Cardiology, Electrotherapy and Angiology, Scanmed, Kraków, Poland; 4 Department of Emergency Medical Services, Faculty of Medicine and Health Sciences Andrzej Frycz Modrzewski Krakow University, Gustawa Herlinga-Grudzińskiego 1, 30-705 Kraków, Poland

**Keywords:** ST-segment elevation myocardial infarctions, Prehospital, First medical contact, System delays, Left ventricular ejection fraction

## Abstract

**Background:**

Cardiovascular disease accounts for nearly half of all deaths in Poland*.* The aim of this study was to assess both the duration and the delays of prehospital treatment in ST-segment elevation myocardial infarction (STEMI) patients and how it impacts left ventricle ejection fraction (LVEF) measured at the time of discharge and the frequency of in-hospital patient mortality.

**Methods:**

This study retrospectively analyzed medical records from January 2011 to December 2015 (excluding the year 2013) of 573 patients who were transported to a hospital with a diagnosis of STEMI.

**Results:**

The mean time of prehospital system delays was 59 min with a maximum time of 152 min and a minimum time of 23 min. The relationship between reduced LVEF (< 55%) and in-hospital patient mortality and the relationship between length of time from first medical contact (FMC) to hospital admission was analysed in 515 respondents. Extending the time of FMC to hospital admission by 1 min increased the chances of lowering LVEF by 2% (95% CI: 1.004–1.041) and increased the chances of death by 2% (95% CI: 1.002–1.04) in STEMI patients.

**Conclusions:**

This study emphasised how vital it is to minimise time spent with STEMI patients at the scene of their cardiovascular event by performing an ECG as quickly as possible and by immediately transporting the patient to the hospital with the targeted treatment. This may lead to the implementation of additional training in the field of ECG interpretation, increase the prevalence of teletransmission systems, and improve communication between Emergency Medical Services (EMS) and catheterization laboratories ultimately reducing patient mortality.

## Background

Cardiovascular disease has accounted for nearly half of all deaths in Poland in the last several decades reaching a total of almost 170,000 deaths annually. Among these deaths, 13,000 are deemed to be caused by an “acute myocardial infarction” [1]. Although this is true, there has been a decrease in the number of STEMIs in Poland in recent years due to the improvement in the quality of diagnosis and care of patients with chest pain. According to the Polish National Registry of Acute Coronary Syndromes (PL-ACS), between 2003 and 2005 the percentage of STEMIs amongst acute coronary syndromes (ACS) was 35–36%. Then a few years later, between 2006 and 2009, it was 30–32% [2]. In 2014, STEMIs accounted for 33% of all ACSs in Poland which received interventional treatment [3].

Studies show that delaying the treatment of myocardial infarction (MI) after the onset of symptoms causes increased mortality. This was confirmed in cases of thrombolytic MI treatment, where the greatest reduction in 35-day mortality was demonstrated in the group of patients in which fibrinolysis occurred within 2 h after the onset of symptoms compared to those treated later (44% vs. 20%, *p* = 0.001) [4]. Studies which analyzed invasive treatment in ACS have also supported the notion of the benefits of rapid intervention. These studies proved that the 30-day mortality of patients was 1% when Percutaneous Coronary Intervention (PCI) was implemented within the first hour of symptoms, 4% when treatment took 60 to 90 min, and 6.4% when the time exceeded 90 min [5]. Additionally, numerous longer observations confirmed this data by showing that every 30 min of delay in treatment resulted in an increase in the risk of death in MI patients by 7.5% annually [6]. For this reason, in accordance with the recommendations of the European Society of Cardiology (ESC) of 2012 as well as of 2017 [7, 8], all hospitals and EMS participating in the treatment of STEMI patients should record and monitor any delay in treatment and strive to achieve the following goals;

- the time from the first medical contact (FMC) to the first ECG performed by the EMS should be ≤10 min in order to quickly diagnose STEMI
- the time from FMC to mechanical reperfusion of the occluded artery should be ≤90 min and in some patients (high-risk patients with a large MI and with symptoms lasting < 2 h) even ≤60 min.

The aim of this study was to assess both the duration and the delays of prehospital treatment in STEMI patients and how it impacts LVEF measured at the time of discharge and the frequency of in-hospital patient mortality.

## Methods

This study was carried out in a center of interventional cardiology and by six EMS teams in the Cracow agglomeration which transported patients to a 24-h catheterization laboratory. Patients who were delivered to the hospital by EMS from the scene of STEMI onset from January 2011 to December 2015 were included in the study (excluding the year 2013). Only patients who were transported to a hospital with a diagnosis of STEMI (534 patients plus 62 patients who were ultimately diagnosed as STEMI ICD10 code: I21.0, I21 or I21.2) were selected for further analysis. Of these 596 STEMI patients, 573 patients were considered for further analysis.

The analysis of medical records was used as the research method in this study. Hospital documentation which included patient history, diagnostic test results, and a EMS ambulance record was retrospectively analysed. The data obtained in the study was collected and stored using Microsoft Office Excel software. Statistical analysis was performed using the Statistica 10.0 program (StatSoft, Inc. STATISTICA software, version 10.0) and the IBM SPSS Software program version 23. A 5% margin of error and a significance level of *p* < 0.05 were accepted. A comparison in the distribution of system delay times in terms of qualitative variables was performed using the U Mann-Whitney test and the Kruskall-Wallis test. The relationship between age, distance, and times was examined by the Spearman’s rank correlation coefficient.

## Results

This study investigated the duration of system delays in the treatment of STEMI patients. System delays are divided into the patient-dependent, the prehospital, and the in-hospital phases. Patient-dependent delay is the time elapsed from the onset of symptoms to the emergency call whose mean in the this study was 71 (24–202) minutes. The maximum time of patient-dependent delay was 6845 min and a minimum of 1 min. Prehospital system delay is the time that expires after a patient calls for the EMS to their hospital admission. This time is dependent on the ambulance and the mean of this delay was 59 (50–72) minutes with a maximum time of 152 min and minimum time of 23 min. The time from admitting the patient to the hospital to mechanical reperfusion of the occluded artery in the catheterization laboratory (door-to-balloon time) is the in-hospital system delay. The mean of in-hospital time was 35 (28–45) minutes with a maximum time of 333 min and a minimum of 11 min.

In order to quantify and measure prehospital delays, the time of the first emergency call, the time of ambulance arrival, the duration of EMS work at the scene, and finally hospital transportation time must all be included in the calculations (Table 1). The mean time it took the EMS to perform an ECG once they were at the scene was 10 (6–18) minutes with a minimum time of 1 min and a maximum time of 85 min. In 50.3% of cases (*n* = 184), the time of performing an ECG by the EMS was in accordance with the ESC guidelines, which recommend the time from the FMC to ECG to be ≤10 min.

**Table 1:** Distribution of Time in System Delays - Prehospital Phase

	Prehospital Phase		
	Time from Emergency Call to FMC (*n* = 573)	Time Spent at the Scene (*n* = 447)	Hospital Transport Duration (*n* = 447)
me (IQR)	10 (7–13) minutes	24 (19–30) minutes	22 (17–28) minutes
min	1 min	1 min	5 min
max	52 min	71 min	89 min

*me (IQR)* interquartile range median, *min* minimum, *max* maximum

In 91.5% of cases (*n* = 515), the door-to-balloon time was in accordance with the ESC guidelines, which recommend that it should last a maximum of 60 min. As part of the assessment of in-hospital delays, the patients’ waiting time for the procedure and the duration of PCI were examined. Delays in the catheterization laboratory are presented in Table 2.

**Table 2:** Distribution of Time in System Delays - Hospital Phase

	Hospital Phase	
	Time to Coronagraphy (*n* = 573)	PCI Duration (*n* = 563)
me (IQR)	15 (11–21) minutes	20 (15–25) minutes
min	1 min	5 min
max	282 min	98 min

*me (IQR)* interquartile range median, *min* minimum, *max* maximum

The time from the FMC to the mechanical reperfusion of the occluded artery in the catheterization laboratory (contact-to-device time) was then calculated. Total ischemic time (TIT) which is the time from the onset of pain to PCI was also determined. The mean time of TIT was 170 (125–300) minute with a maximum time of 6968 min and a minimum time of 48 min.

The mean time from FMC (EMS arrival at the site) to PCI was 87 (74–103) minutes with a maximum time of 395 min and a minimum of 38.5 min. The ESC recommendations regarding the time from FMC to mechanical reperfusion of the occluded artery of ≤90 min was met in 55.6% of cases (*n* = 313). However, for 368 patients with symptoms lasting less than 2 h, the contact-to-device recommendation time of ≤60 min was met only in 6.8% (*n* = 25).

### The influence of the duration of prehospital treatment on the value of the maximum left ventricle ejection fraction and in-hospital death

Echocardiography with the assessment of maximal LVEF was performed in 552 patients (96.3%). The comparison between people who had normal maximum LVEF (LVEF ≥55%) and those who had it reduced (LVEF < 55%) are presented in Table 3. There is no difference in the mean age between people with normal maximum LVEF and those with lowered LVEF. The mean distance from the place where the patient was taken to the hospital is higher by 2.2 km in people with LVEF < 55%. The mean time from FMC to handing over the patient to the hospital was significantly longer (by 5 min) in people with reduced ejection fraction. There was no significant difference in the time from first entering the ambulance to PCI implementation between people with normal and reduced LVEF.

**Table 3:** Comparison of Variables Between Subjects with Normal LVEF (≥55%) and Reduced LVEF(< 55%)

Variable	LVEF	n	x	Sd	me	IQR	*p*
Age [years]	≥ 55%	74	63.6	10.2	**–**	**–**	0.6^A^
	< 55%	478	64.4	11.9	**–**	**–**	
Distance from Hospital [km]	≥ 55%	74	**–**	**–**	8.2	3.2–19.2	0.01^B^
	< 55%	478	**–**	**–**	11.1	5.4–23.4	
Time from FMC to Hospital Admission [min]	≥ 55%	74	**–**	**–**	44	36–54	0.005^B^
	< 55%	478	**–**	**–**	49	41–61	
Time from FMC to PCI [min]	≥ 55%	74	**–**	**–**	84	70–95	0.076^B^
	< 55%	478	**–**	**–**	87	75–103	

*LVEF* left ventricular ejection fraction, *n* sample, *x* sample mean, *Sd* standard deviation, *me* mean, *IQR* interquartile range, *p p* value

A - *p* value from t-Student test

B - *p* value from U Mann-Whitney test

In-hospital deaths, 38 out of 573 patients (6.7%), occurred most frequently in the first day of hospitalization (52.6%). A comparison between people who died in the hospital and those discharged from the hospital is presented in Table 4. The average age of patients who died in the hospital was 6.3 years higher than those discharged from the hospital. There was no significant difference in the mean distance from where the patient was first taken to the hospital between those who died and those who had been discharged from the hospital. There was no significant difference in time from the arrival of the ambulance to the site of the event to hospital admission between people who died and those who did not. The mean time from FMC to the mechanical reperfusion of the occluded artery in people who died was significantly longer (by 9 min) than in those patients who were discharged from the hospital.

**Table 4:** Comparison of Variables Between Patients Who Died in the Hospital and Those Discharged from the Hospital

Variable	Death?	n	x	Sd	me	IQR	*p*
Age [years]	no	535	63.9	11.6	**–**	**–**	0.001^A^
	yes	38	70.3	13.9	**–**	**–**	
Distance from Hospital [km]	no	535	**–**	**–**	10.30	5.2–22.1	0.09^B^
	yes	38	**–**	**–**	6.80	2.9–18.0	
Time from FMC to Hospital Admission [min]	no	535	**–**	**–**	48	41–60	0.2^B^
	yes	38	**–**	**–**	51.5	44–62	
Time from FMC to PCI [min]	no	525	**–**	**–**	86	74–101	0.005^B^
	yes	34	**–**	**–**	105	79–118.5	

*LVEF* left ventricular ejection fraction, *n* sample, *x* sample mean, *Sd* standard deviation, *me* mean, *IQR* interquartile range, *p p* value

A - *p* value from t-Student test

B - *p* value from U Mann-Whitney test

In the group of patients in whom PCI was performed within 60 min after hospital admission, the influence of prehospital treatment ‘s duration on the value of the maximum LVEF and in-hospital death was evaluated. Among the 515 respondents meeting this criteria, 432 (83.9%) patients had a reduced maximum LVEF and 38 (6.7%) of the respondents died in the hospital. A one-dimensional model showed that prolonging the time from FMC to patient hospital admission significantly influenced the chance of a reduced LVEF value and in-hospital death. Extending this time by 1 min increased the chances of lowering LVEF by 2% (95% CI: 1.004–1.041) and increased the chances of death by 2% (95% CI: 1.002–1.04).

Additionally, multivariable regression models were performed for all pre-hospital procedures tested. The results in these models include the administration of antiplatelet/ anticoagulant drugs (heparin, clopidogrel or both) because they have been significantly associated with decreased left ventricular contraction in previously performed multivariable models. After taking into account the influence of age, sex, presence of diabetes, hypertension, hypercholesterolemia, renal failure, shock and the administration of drugs (heparin, clopidogrel), the overall effect the duration of time from FMC to patient hospital admission on LVEF and in-hospital death was decreased and became statistically insignificant (Tables 5 and 6).

**Table 5:** The Relationship Between the Occurrence of Reduced LVEF and the Time from FMC to Patient Hospital Admission - One-Dimensional and Multivariable Model

	OR^a^	95% CI		OR^b^	95% CI	
Time from FMC to Patient Hospital Admission	1.022	1.004	1.041	1.017	0.994	1.042

*OR* Odds Ratio

*CI* Confidence Interval

^a^ one-dimensional model

^b^ multivariable model including drugs + age + sex + distance + diabetes + hypertension + hypercholesterolemia + renal failure + shock

**Table 6:** The Relationship Between the Occurrence of In-Hospital Death and the Time from FMC to Patient Hospital Admission - One-dimensional and Multivariable Model

	OR^a^	95% CI		OR^b^	95% CI	
Time from FMC to patient hospital admission	1.022	1.002	1.04	1.005	0.976	1.035

*OR* Odds Ratio

*CI* Confidence Interval

^a^ one-dimensional model

^b^ multivariable model including drugs + age + sex + distance + diabetes + hypertension + hypercholesterolemia + renal failure + shock

## Discussion

The aim of this study was to assess both the duration and the delays of prehospital treatment in STEMI patients and how it impacts LVEF measured at the time of discharge and the frequency of in-hospital patient mortality. Delays in prehospital treatment for patients with an MI consist of patient-dependent delays and system delays. System delays are determined by the ambulance that transports the patient and in part by the hospital that implements the final treatment of PCI. The reduction of the time from the onset of symptoms to reperfusion therapy is a priority in the treatment of patients with MI.

The time after a MI patient comes into contact with health services is the component of time which depends solely on the healthcare system. This time before PCI is influenced by two independent phases which are the pre-hospital phase and catheterization laboratory phase. So far, the health care system has focused on shortening the time from the moment of admitting the patient to the hospital and performing PCI (door-to-balloon time) keeping this as a determinant of good care for a patient with a MI.

Door-to-balloon time did initially compose the majority of the delays seen in patients with a MI. In 27,080 patients analysed in 661 centers in the USA in the years between 1994 and 1998, the mean time from first symptoms to hospital admission was 96 min. While the mean time from hospital admission to PCI (door-to-balloon) was 116 min (IQR 85–163) [9]. This makes it very clear that reducing door-to-balloon time was the national target for the treatment of heart attacks in the United States. The 2013 American Heart Association (AHA) guidelines recommended keeping this time below 90 min. Furthermore, the 2012 ESC guidelines proposed that hospitals which can perform PCI should seek to reduce door-to-balloon time to ≤60 min. Once these recommendations were put into place, in-hospital delays have been significantly reduced. In the US in 2005, the door-to-balloon mean time was 86 min (IQR 65–109), and in 2011 it dropped to 63 min (IQR 47–80) [10]. In Poland between 2003 and 2006 (from October 2003 to March 2006), the mean time of door-to-balloon was 50 min (IQR 32–85) [11] and in 2009 it was 25 min (PL-ACS) [2]. In this study, the mean time of door-to-balloon was 35 (28–45) minutes. This result is in line with both AHA and European Resuscitation Council recommendations.

When focusing on total time from FMC to PCI, Poland had comparable results to other European nations. According to PL-ACS reports between 2004 and 2007, the mean time from FMC to PCI was 124 min in Poland [12]. In Germany, the Czech Republic, Croatia, and Lithuania, the time from FMC to PCI was 120 min. The shortest times were reported in Belgium (60 min) and in Sweden (69 min). The longest times reported were in France (170 min) and Serbia (177 min) [12]. Among 3312 patients with STEMI in Cologne, Germany in the years between 2006 and 2012, the mean time from FMC to PCI was 65 min (IQR 48–91) when the patient was transported directly to the hospital where PCI was performed. This time was 89 min (IQR 72–115) when the patient was brought by ambulance and 107 min (IQR 85–148) when the patient was transported from another hospital [13]. In Cracow in 2009, the time from FMC to PCI was on average 93 min in patients who were directly transported to the catheterization laboratory. In cases where the patient was transported from hospital emergency departments to catheterization laboratories, the average time was between 175 min (in Cracow) and 193 min (outside of Cracow) [14]. In Warsaw, the mean time of contact-to-device time was 159 min in patients transported from other hospitals. This time decreased to 115 min in patients who were brought directly to the site of PCI after the EMS consulted the catheterization laboratory with ECG teletransmission [15]. In this study, the mean time from FMC to PCI was 87 (74–103) minutes. This evidence makes it clear that correctly transporting patients to the appropriate location is crucial in order to diminish delays. It is worth noting, though, that the time of FMC in other studies was chosen differently. In some studies, FMC was noted at the time of arrival of a medical rescue team at the place of the cardiovascular event [16, 17]. In others, FMC was the time the ECG was performed by the EMS [18, 19, 20]. Moreover, other studies used the time of the patient’s phone call to the EMS as FMC [21]. Using the time patients called the ambulance as FMC may be unreliable because that phone call would be the only way to assess the patient’s condition. In this study, STEMI was confirmed by ECG in patients with a history of chest pain but the exact time of recording was not able to be assessed in 36.1% of cases. For this reason, the decision was made to include the travel time of EMS in the time of FMC. Therefore, the diagnosis of STEMI by ECG is by definition the moment of FMC which is in accordance with the ESC guidelines of 2017. Unification of these definitions could help guide both the monitoring and comparisons made between different centers in the quality of STEMI patient care.

Door-to-balloon time focuses only on the last phase of treatment in STEMI patients. This study proved that with modifications in earlier phases, it is possible to improve the care of STEMI patients. The influence of how fast EMS procedures were performed on STEMI patients on the outcomes of the final treatment was assessed. The one-dimensional model in this study showed that prolonging the time from FMC to patient hospital admission significantly influenced the chance of a reduced LVEF value and in-hospital death, though in the multivariable model this relationship did not maintain its significance. Longer durations of the prehospital phase was directly correlated with greater distances in which some patients had to travel in order to reach the hospital. In this study, patients who had to travel greater distances resulted in the reduction of LVEF in those analyzed patients. Patients who came from greater distances, though, had an unchanged door-to-balloon time as in all those cases the PCI team was notified in advance of the patient’s arrival and was adequately prepared.

A review in Denmark with a group of 6209 patients, observed a significant increase in mortality with an increase in system delays times (> 1 h 15.4% vs. 1-2 h 23.3% vs. 2-3 h 30.8%, p > 0.001) [22]. Also, Koul et al. showed a significant relationship between FMC to PCI delay and one-year mortality in a group of 13,790 patients. In their work, extending system delay time over 1 h resulted in a significant increase in the chance of death by 26% (OR = 1.26, 95% CI: 1.03–1.55). With a delay of more than 2 h, this chance of death was even increased by 51% (95% CI: 1.23–1.86) [23].

This study, in accordance with others similar works, showed that the duration of system delays, between FMC and PCI, was directly tied to an increased chance in in-hospital patient death. As previously stated, shortening system delays may be achieved by notifying the PCI team in advance. Additionally, other sources note that by omitting the emergency department and directly sending the patient to the invasive cardiology department also shortens system delays [24].

Focus should be placed on minimizing delays in the prehospital phase as this is the longest delay in the healthcare of STEMI patients as noted in this study and confirmed in others. Literature on this matter notes that the best way to accelerate the diagnosis of STEMI in a patient, consequently shortening system delays, is by performing an ECG faster after FMC. In Germany, the mean time of performing an ECG after FMC was 5 min (IQR 3-10 min) [25]. Furthermore, it would be beneficial to minimize time spent with the patient at the scene of cardiac event in order to transport the patient to the hospital more quickly where PCI may be performed.

Ultimately, one of the most crucial elements in shortening system delays can be found in ECG interpretation and the physical exam. The ability to assess ECGs by various groups of medical professions has been evaluated in several studies. In one study in a group of paramedics, the correct diagnosis of myocardial infarction ranged from 59 to 94%. In a group of emergency medicine doctors, a correct diagnosis was seen in 77 to 93% of the time, and finally in a group of cardiologists, the correct diagnosis was established in up to 81 to 95% of cases [26, 27, 28]. ECG teletransmission to the catheterization laboratory is a key piece in shortening delays. In Poland, the teletransmission system has been developing since 2001 and is now available in almost 100% of State Emergency Medical Service Ambulances. In Cracow, Poland, it has been accessible since 2013. However, teletransmissioncannot ultimately replace the knowledge and competence of EMS members. This is because there are sometimes situations when either human error, equipment failure, or lack of Global System for Mobile communications may make teletransmission impossible to perform.

Currently, the consensus among medical practitioners is that the preferred method of STEMI treatment is PCI. The European Society of Cardiology guidelines of 2018, however, also addresses the possibility of implementing fibrinolysis by paramedics in pre-hospital conditions in cases of prolonged transport. Fibrinolytics are therefore supplied by ambulances in several European and in Northern American countries [29, 30]. The European Society of Cardiology guidelines of 2018 suggest that in situations when the maximum time from diagnosis of STEMI to primary PCI is above 120 min, fibrinolysis should be considered and, if possible, used in the pre-hospital setting [8]. Administering such medications shortens the time of opening the occluded artery and is especially useful in rural areas [31]. However, medical emergency personnel in Poland are not legally able to administer fibrinolytics. Poland, though, has a dense network of catheterization laboratories (165 for a population of 38.5 million), and in most cases, the time from diagnosis of STEMI to primary PCI is less than 120 min.

## Conclusions

In conclusion, focusing on the time of FMC with the health care system to the start of reperfusion therapy is important because it takes into account the total time of delays which are modifiable by the health care system. Understanding the weak elements of this “survival chain” may allow for the implementation of appropriate changes which may lead to more improvements in the care of STEMI patients. In this study, the longest system delay was seen in the time of prehospital procedures. After conferring this data with other studies, this study suggests that it is possible for EMS to accelerate the diagnosis of STEMI by performing ECGs faster after FMC. The goal should be to minimize the time spent with patients at the site of the event and perform an ECG as quickly as possible in order to transport the patient more efficiently to the hospital with the targeted treatment (PCI). This time depends partly on the patient’s condition as well as on the distance to the catheterization laboratory. It is therefore important to remind members of the EMS in meetings or in ECG interpretation trainings on the key role time plays while treating patients with STEMI.

## Abbreviations

ACS: acute coronary syndromes
AHA: American Heart Association
EMS: Emergency Medical Services
ESC: European Society of Cardiology
FMC: first medical contact
LVEF: left ventricle ejection fraction
MI: myocardial infarctio
PCI: Percutaneous Coronary Intervention
PL-ACS: Polish National Registry of Acute Coronary Syndromes
STEMI: ST-segment elevation myocardial infarction
TIT: total ischemic time

## References

[1] Central Statistical Office (2016). Demographic yearbook 2016.

[2] Poloński L, Gąsior M, Gierlotka M (2011). What has changed in the treatment of ST-segment elevation myocardial infarction in Poland in 2003-2009? Data from the polish registry of acute coronary syndromes (PL-ACS). Kardiol Pol.

[3] Ochała A, Siudak Z, Legutko J (2015). Percutaneous interventions in cardiology in Poland in 2014. Report of the Board of Association of cardiovascular interventions of the polish cardiac society (AISN PTK). Kardiol Pol.

[4] Boersma E, Maas AC, Deckers JW (1996). Early thrombolytic treatment in acute myocardial infarction: reappraisal of the golden hour. Lancet..

[5] A clinical trial comparing primary coronary angioplasty with tissue plasminogen activator for acute myocardial infarction. The global use of strategies to open occluded coronary arteries in acute coronary syndromes (GUSTO IIb) angioplasty substudy investigators. N Engl J Med 1997;336:1621–1628. 10.1056/NEJM199706053362301. 10.1056/NEJM199706053362301 9173270

[6] De Luca G, Suryapranata H, Ottervanger JP (2004). Time delay to treatment and mortality in primary angioplasty for acute myocardial infarction: every minute of delay counts. Circulation..

[7] Steg G, James SK, Atar D, et al. ESC guidelines for the management of acute myocardial infarction with persistent ST segment elevation. Kardiol Pol 2012; 70, VI: 255–318.

[8] Ibanez, B., James, S., Agewall, S., et al. (2019). 2017 ESC Guidelines for the management of acute myocardial infarction in patients presenting with ST-segment elevation. Oxford Academic. Available at: https://academic.oup.com/eurheartj/article/39/2/119/4095042. [Accessed 23 Feb. 2019]. 10.1016/j.rec.2017.11.010 29198432

[9] Cannon CP, Gibson CM, Lambrew CT (2000). Relationship of symptom-onset-to-balloon time and door-to-balloon time with mortality in patients undergoing angioplasty for acute myocardial infarction. JAMA..

[10] Nallamothu BK, Normand SL, Wang Y (2015). Relation between door-to-balloon times and mortality after primary percutaneous coronary intervention over time: a retrospective study. Lancet..

[11] Poloński L, Gasior M, Gierlotka M (2007). Polish registry of acute coronary syndromes (PL-ACS) characteristics, treatments and outcomes of patients with acute coronary syndromes in Poland. Kardiol Pol.

[12] Widimsky P, Wijns W, Fajadet J (2010). Reperfusion therapy for ST elevation acute myocardial infarction in Europe: description of the current situation in 30 countries. Eur Heart J.

[13] Pfister R, Lee S, Kuhr K (2016). Impact of the type of first medical contact within a guideline-conform ST-elevation myocardial infarction network: a prospective observational registry study. PLoS One.

[14] Dudek D, Legutko J, Siudak Z (2010). Organization of interventional treatment of patients with STEMI and NSTEMI heart attack in Poland. Kardiol Pol.

[15] Karcz Maciej, Bekta Pawel, Skwarek Miroslaw, Dabrowski Maciej, Kukula Krzysztof, Kadziela Jacek, Malek Lukasz, Ciszewski Michal, Debski Artur, Ruzyllo Witold (2009). P-392 By-Passing Non-PCI Hospitals Following ECG Transmission Coupled with On-Site Interventionalist Duty Minimises Delay to Primary PCI. CVD Prevention and Control.

[16] Mumma BE, Kontos MC, Peng SA (2014). Association between prehospital electrocardiogram use and patient home distance from the percutaneous coronary intervention center on total reperfusion time in ST-segment-elevation myocardial infarction patients: a retrospective analysis from the national cardiovascular data registry. Am Heart J.

[17] Kleinrok A, Płaczkiewicz DT, Puźniak M (2014). Electrocardiogram teletransmission and teleconsultation: essential elements of the organisation of medical care for patients with ST segment elevation myocardial infarction: a single Centre experience. Kardiol Pol.

[18] Adams R, Appelman Y, Bronzwaer JG (2010). Implementation of a prehospital triage system for patients with chest pain and logistics for primary percutaneous coronary intervention in the region of Amsterdam, the Netherlands. Am J Cardiol.

[19] Sillesen M, Sejersten M, Strange S (2008). Referral of patients with ST-segment elevation acute myocardial infarction directly to the catheterization suite based on prehospital teletransmission of 12-lead electrocardiogram. J Electrocardiol.

[20] Martinoni A, DeServi S, Boschetti E (2011). Importance and limits of pre-hospital electrocardiogram in patients with ST elevation myocardial infarction undergoing percutaneous coronary angioplasty. Eur J Cardiovasc Prev Rehabil.

[21] Ho AF, Pek PP, Fook-Chong S (2015). Prehospital system delay in patients with ST-segment elevation myocardial infarction in Singapore. World J Emerg Med.

[22] Terkelsen CJ, Sørensen JT, Maeng M (2010). System delay and mortality among patients with STEMI treated with primary percutaneous coronary intervention. JAMA..

[23] Koul S, Andell P, Martinsson A (2014). Delay from first medical contact to primary PCI and all-cause mortality: a nationwide study of patients with ST-elevation myocardial infarction. J Am Heart Assoc.

[24] Kawakami S, Tahara Y, Noguchi T (2016). Time to reperfusion in ST-segment elevation myocardial infarction patients with vs. without pre-hospital Mobile telemedicine 12-Lead electrocardiogram transmission. Circ J.

[25] Zeymer U, Arntz HR, Dirks B (2009). Reperfusion rate and inhospital mortality of patients with ST segment elevation myocardial infarction diagnosed already in the prehospital phase: results of the German prehospital myocardial infarction registry (PREMIR). Resuscitation..

[26] Huitema AA, Zhu T, Alemayehu M (2014). Diagnostic accuracy of ST-segment elevation myocardial infarction by various healthcare providers. Int J Cardiol.

[27] Feldman JA, Brinsfield K, Bernard S (2005). Real-time paramedic compared with blinded physician identification of ST-segment elevation myocardial infarction: results of an observational study. Am J Emerg Med.

[28] Sejersten M, Young D, Clemmensen P (2002). Comparison of the ability of paramedics with that of cardiologists in diagnosing ST-segment elevation acute myocardial infarction in patients with acute chest pain. Am J Cardiol.

[29] Björklund E (2006). Pre-hospital thrombolysis delivered by paramedics is associated with reduced time delay and mortality in ambulance-transported real-life patients with ST-elevation myocardial infarction. Eur Heart J.

[30] Welsh RC (2006). Feasibility and applicability of paramedic-based prehospital fibrinolysis in a large north American center. Am Heart J.

[31] Bergmeijer TO (2013). Prehospital treatment of ST-segment elevated myocardial infarction patients. Futur Cardiol.

